# Effect of Chin‐down‐plus‐larynx‐tightening maneuver on swallowing function after minimally invasive esophagectomy: A randomized controlled trail

**DOI:** 10.1002/cam4.3280

**Published:** 2020-07-06

**Authors:** Funa Yang, Limin Zou, Lijuan Li, Qiyun Zou, Peinan Chen, Haibo Sun, Xianben Liu, Xiaoxia Xu

**Affiliations:** ^1^ Nursing Department Affiliated Cancer Hospital of Zhengzhou University Henan Cancer Hospital Zhengzhou China; ^2^ Department of Thoracic Surgery The Affiliated Cancer Hospital of Zhengzhou University Zhengzhou China

**Keywords:** Chin‐down‐plus‐larynx‐tightening maneuver, esophageal cancer, esophagectomy, swallowing function

## Abstract

**Background:**

The incidence of swallowing abnormality was high after minimally invasive esophagectomy (MIE) for esophageal cancer (EC). Few reports, however, focused on interventions for dysphagia after esophagectomy.

**Aim:**

The purpose of this research was to estimate the effect of Chin‐down‐plus‐larynx‐tightening maneuver on swallowing function for patients receiving esophagectomy.

**Method:**

This was a 2‐arm, parallel‐group, single‐blind randomized clinical trial, performed in patients suffered from EC from November 2018 to January 2020. Patients were randomly assigned to the intervention group (IG) or the control group (CG). The participants in CG received routine care, and the IG received Chin‐down‐plus‐larynx‐tightening maneuver during feeding. The incidence of choking cough, swallowing function, and dietary outcomes were evaluated before and after intervention for 7 days.

**Results:**

A total of 237 EC cases were enrolled and randomized to the IG (n = 118) or CG (n = 119). There was no significant difference between the two groups in terms of demographic and clinical characteristics. Postoperative choking cough occurred in 5 of 118 cases (4.24%) in IG and 18 of 119 cases (19.4%) in CG, the differences showed statistically significant (*P* < .001). The analysis showed that the participants in the IG compared with the CG have more total caloric intake of 24 hours and higher *K*/*R* (the ratio of calories oral achieved to total calories required of body) significantly from D1 to D7 of intervention (*P *< .05).

**Conclusion:**

The findings suggest that the Chin‐down‐plus‐larynx‐tightening maneuver can improve swallowing function recovery and oral total food intake and calories in EC patients undergoing MIE.

## Graphical abstract

The incidence of swallowing abnormality was high after esophagectomy, which affects patients' nutritional intake and quality of life. The Chin‐Down maneuver can achieve good results in improving swallowing performance caused by Stroke, Parkinson and other diseases. In this study, we hypothesized that changing the position of head and body might affect the swallowing parameters and improve swallowing abnormalities in patients after esophagectomy, which we called it as the Chin‐down‐plus‐larynx‐tightening maneuver. The findings suggest that the maneuver can improve swallowing function recovery and oral total food intake and calories in patients undergoing esophagectomy.

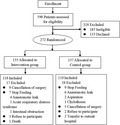

## 1 INTRODUCTION

Esophageal cancer (EC) can be responsible for an estimated 1 in every 20 cancer deaths in 2018, which ranks seventh in terms of incidence (572 000 new cases) and sixth in mortality overall (509 000 deaths) on the global burden of cancer worldwide.^1^ The incidence of EC shows obvious geographic differences, about 80% cases occurring in developing countries.^2^ It is estimated that around 165 000 new cases of EC occur each year in China.^3^ Surgery is the standard treatment for resectable EC, and approximately half of all EC surgeries in the world are performed in China.^3^

McKeown minimally invasive esophagectomy (MIE) is the main surgical method, which has been performed at our institution since 2007. However, the incidence of swallowing abnormality was high after operation, since following factors^4^: (a) the high position of left neck anastomosis during operation; (b) the injuries or paralysis of the recurrent laryngeal nerve due to dissection of lymph node; (c) impaired epiglottis eversion, pharyngeal palsy, incomplete laryngeal elevation caused by tracheal intubation, which mainly manifested as aspiration, choking cough, and hoarseness. Aspiration and choking cough has been occurred in 33%^5^ and 53.2%,^6^ respectively, after resectable thoracic EC.

Short‐ or long‐term nutritional risk is a common problem for patients undergoing esophagectomy. Study has shown that enteral nutrition can be performed within 24 hours after surgery,^7^ which could significantly decrease the pulmonary complications, anastomotic leakage, and maintain a better nutritional status for patients.^8^, ^9^, ^10^ However, patients' total caloric intake can only meet approximately 75% of their postoperative energy requirements, one of the important factors is delayed oral food intake after surgery.^11^ When patients face problems such as choking cough and swallowing disability, patients often struggle to establish oral nutritional intake due to fear, gastrointestinal symptoms, and even pneumonia. The nasointestinal or jejunostomy tubes will replace oral feeding for enteral nutrition, which will impair patients' quality of life seriously. Therefore, how to improve the swallowing function and reduce the incidence of choking cough effectively are particularly crucial for patients following esophagectomy.

At present, there are many intervention studies on dysphagia related to stroke, Parkinson and other diseases, including expiratory muscle strength training, surface electrical stimulation, biofeedback therapy, and onmyofunctional exercises for swallowing.^12^, ^13^ However, few studies focus on interventions for dysphagia after esophagectomy. Kumai et al^4^ collected a prospective data from patients who had normal swallowing function after an esophagectomy examined by videofluoroscopic and found that head and body positions can affect swallowing patterns. Annelise et al^14^ found that the chin‐down maneuver can achieve good results in improving swallowing performance and self‐perception of Parkinson's disease patients. Yu et al^6^ observed 22 patients who choked on water after EC surgery and guided them to swallow water using Chin‐down‐plus‐larynx‐tightening maneuver, the research confirmed the new maneuver can help manage choking cough.

In this study, we hypothesized that changing the position of head and body might affect the swallowing parameters and improve swallowing abnormalities in patients after esophagectomy. We investigated the utility of a novel maneuver prompted by anecdotal clinical observations, combining the chin‐down with larynx‐tighten, which was defined as Chin‐down‐plus‐larynx‐tightening maneuver. The standard operating procedures has been developed. The randomized controlled trial has been conducted to evaluate the role of Chin‐down‐plus‐larynx‐tightening maneuver in improving the recovery of swallowing function in EC patients after surgery, and thus could provide a reference for clinical nursing work.

## 2 METHODS

### 2.1 Research design

This was a 2‐arm, parallel‐group, single‐blind randomized clinical trial, performed in patients with EC who had been treated with esophagectomy. Written informed consent was obtained from all participants or their family members before the trial. In addition, the principles of the Helsinki declaration have been respected.

### 2.2 Randomization and masking

The patients were assigned to the intervention and control groups randomly with standard computerized randomization algorithms. Given the nature of intervention research, it was not feasible to impose blindness on researchers and interveners. However, all patients, research assessors, and data management staff were kept strictly blinded to trial design and outcome measurements. Furthermore, in order to avoid mutual contamination between patients, we tried to place patients in different areas of the ward, patients and families were asked to avoid discussing their interventions with each other.

### 2.3 Inclusion and exclusion criteria

The study was conducted at the Department of Thoracic Surgery of our hospital in China, where approximately 900 patients undergoing esophagectomy annually. Patients with EC who had received surgery were recruited from November 2018 to January 2020. All subjects should meet the following conditions. Inclusion criteria: (a) diagnosed with carcinoma of the thoracic esophagus based on pretreatment biopsy samples; (b) age ≤ 70 years; (c) undergoing McKeown minimally invasive esophagectomy; (d) Patients informed consent and volunteered to this study. Exclusion criteria included: (a) emergency or open surgery; (b) undergoing radiotherapy before surgery; (c) with severe complications which may cause patients to stop feeding such as anastomotic leakage; (d) merged with Parkinson's disease, cerebral infarction, and other serious organic diseases; (e) a documented history of schizophrenia or psychosis; (f) inability to perform language communication or text understanding.

### 2.4 Interventions

Both groups received the same minimally invasive surgery and treated similarly in the perioperative period. The feeding protocol for all patients is as follows: At POD1, the gastric tube was pulled out, patients were only allowed to drink water; at POD 2, liquid food was administered, such as rice soup; a semiliquid diet was allowed at POD 3; then, soft solid food, such as steamed bread was allowed. Trained nurses provided diet guide and education for all patients.

#### 2.4.1 The Intervention Group (IG) (Chin‐down‐plus‐larynx‐tightening maneuver Group)

**Figure 1:**
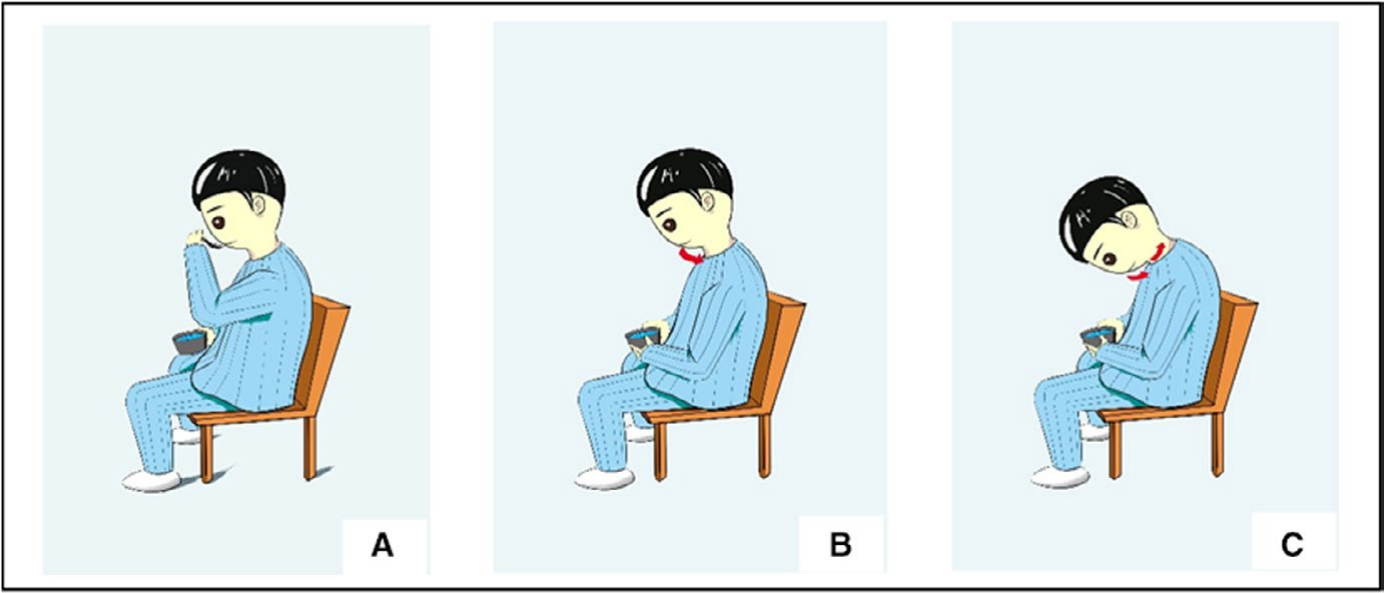
The process and postures of Chin‐down‐plus‐larynx‐tightening maneuver (A) Patient drunk 5 mL warm water with spoon; (B) Water was retained in the mouth and head was bowed slowly; (C) The chin was moved as close to sternum as possible, and the larynx was tightened toward back (red arrow)

1. Item preparation: a cup with measuring scale, some warm water, a spoon, a small table board on the bed.
2. Posture preparation: Participants took standing or sitting position, keeping their torso upright, chest up and tight, and shoulders relaxed. Head and neck relaxation training were given before exercising.
3. Chin‐down‐plus‐larynx‐tightening maneuver: Participants were instructed to hold 5 mL of warm water in their mouth by spoon, move the chin as close to the sternum as possible, tighten the larynx, and then swallow the water slowly. The process and postures of Chin‐down‐plus‐larynx‐tightening maneuver are shown in Figure 1. The participants are required to focus during exercise. Trained nurse observed and recorded any discomfort throughout the process.
4. Timing of exercise:
  - The first day before operation. Nurse began to instruct participants to perform Chin‐down‐plus‐larynx‐tightening maneuver exercise twice (10 maneuver each time);
  - Attempting to drink water (usually at the afternoon of the first day after surgery). A trained nurse prepares the items, assisted patients to sit up on the bed, instructed patients to swallow using the Chin‐down‐plus‐larynx‐tightening maneuver, and observes patient's response; If participant had a persistent, difficult‐to‐relieve cough after drinking, oral intake was stopped immediately, and doctor will be reported. If no abnormality was observed, exercise continued again after one hour.
  - Individuals were allowed to drink more water and increase food intake gradually, from liquid to semi‐liquid and normal diet. This maneuver was repeated for every swallow until patients were discharged.
  - Precautions: Choking cough mainly occurs at the beginning of drinking water after surgery, so nurse should provide guidance at the bedside during the whole process to ensure patients' safety. Put small portions of food in mouth and swallow slowly. Take another bite until there was no residue in mouth. Talking was not allowed during feeding. Chew the food well before swallowing. Avoid lying down right after the finish of a meal, stay seated for 30 minutes or get out of bed for at least 15 minutes to prevent food reflux.

All instructions, as well as explanations about the maneuver, were submitted to the patients through a written document.

#### 2.4.2 The Control Group (CG)

The patients in the CG ate as usual, with their neck and head in any comfortable position. Their eating process was observed and recorded by researcher. In addition, patients also received routine nursing measures after McKeown minimally invasive esophagectomy, including a safe and comfortable environment, diet guidance, postoperative rehabilitation exercises, medication care, psychological counseling and so on.

### 2.5 Assessments and sample size

At baseline, all patients underwent preoperative assessment before intervention (the first time to drink water), including the incidence of choking cough, swallowing function. Subsequently, patients were reassessed after 7 days of intervention (because patients are usually discharged at 8‐9 days postoperative). To ensure the accuracy of assessment, we make the following efforts: First, every subject received symptom evaluation twice, combining the situation of surgery and treatment; Second, two‐person assessment was performed including a doctor and a nurse; Third, when the patient coughs during drinking or eating, we will conduct an evaluation again after 30 minutes under the supervision of medical staff.

The evaluation indicators of this study were as follows: (a) Incidence of choking cough: In this study, we defined choking cough as an irritating cough that occurred immediately when patients began drinking or eating. When two consecutive coughs occurred during drinking or eating, we considered it to occur. The incidence of choking cough = the number of patients with cough/ total number of observers. (b) Swallowing function, as measured by the Kubota water‐drinking test^15^ before and after intervention. This test required participants attempt to drink 30 mL of warm water, measuring the time required to finish drinking and observing any choking. The swallowing function was divided into five levels based on swallowing movements and accompanying cough. Level Ⅰ: Swallowing water smoothly once within 5 seconds without choking; Level Ⅱ: Swallowing water once within 5 seconds with choking, or swallowing twice in more than 5 seconds without choking; Level Ⅲ: Swallowing water once successfully in more than 5 seconds with choking; Level Ⅳ: Drinking was finished by swallowing more than twice in more than 5 seconds with choking; Level Ⅴ: Frequent coughing and difficulty in swallowing all the water.^3^ Dietary outcomes: Nurses calculated caloric requirements of patients based on the modified Harris‐Benedict formula + 30% for postoperative energy requirements.^16^ Nurses were responsible for observing and recording the type and amount of food patients eat at each meal. Nutritionist was in charge of calculating the daily calorie intake of patients. Dietary outcomes contained the total caloric intake and *K*/*R* value (the ratio of calories oral achieved to total calories required of body) from D1 to D7 of intervention.

The incidence of postoperative choking cough was the main outcome measure for this study. Based on the previous research^17^ (3.39% in the IG and 14.52% in the CG), the sample size was estimated according to the degree of certainty 1−*β* = 0.80 and the test level α = 0.05, by using PASS 8.0 software. The formula is as follows:

$$n_{1}\;=\;n_{1}\;=\;\frac{{\left[\mu_{\alpha }\sqrt{2p(1‐p)}\;+\;\mu_{\beta }\sqrt{p_{1}\left(1‐p\right)+p_{2}\left(1‐p_{2}\right)}\right]}^{2}}{{\left(p_{1}‐p_{2}\right)}^{2}}.$$

In the formula, p1 and p2 are estimated values of the incidence of cough in the intervention and control groups, respectively, and p is the combined incidence of two groups. We calculated the sample size to be 206 (103 participants per group). Allowing for 10% attrition, we increased the sample size to 230 patients (115 participants per group) at baseline.

### 2.6 Statistical analysis

Descriptive statistics were used for patient demographic and clinical characteristics at baseline. Continuous variables were presented as Means ± SD and compared using the unpaired t test if the distribution was normal or presented as medians (IQR) and compared using the Mann‐Whitney *U* test if the distribution was non‐normal. Categorical or ranked variables were presented as frequency (%), and were analyzed with the *χ*2 or Fisher exact test. *P* < .05 was considered statistically significant. The statistical analysis was performed using SAS 9.4 (SAS Institute Inc).

## 3 RESULTS

### 3.1 Participant demographics and clinical characteristics

**Figure 2:**
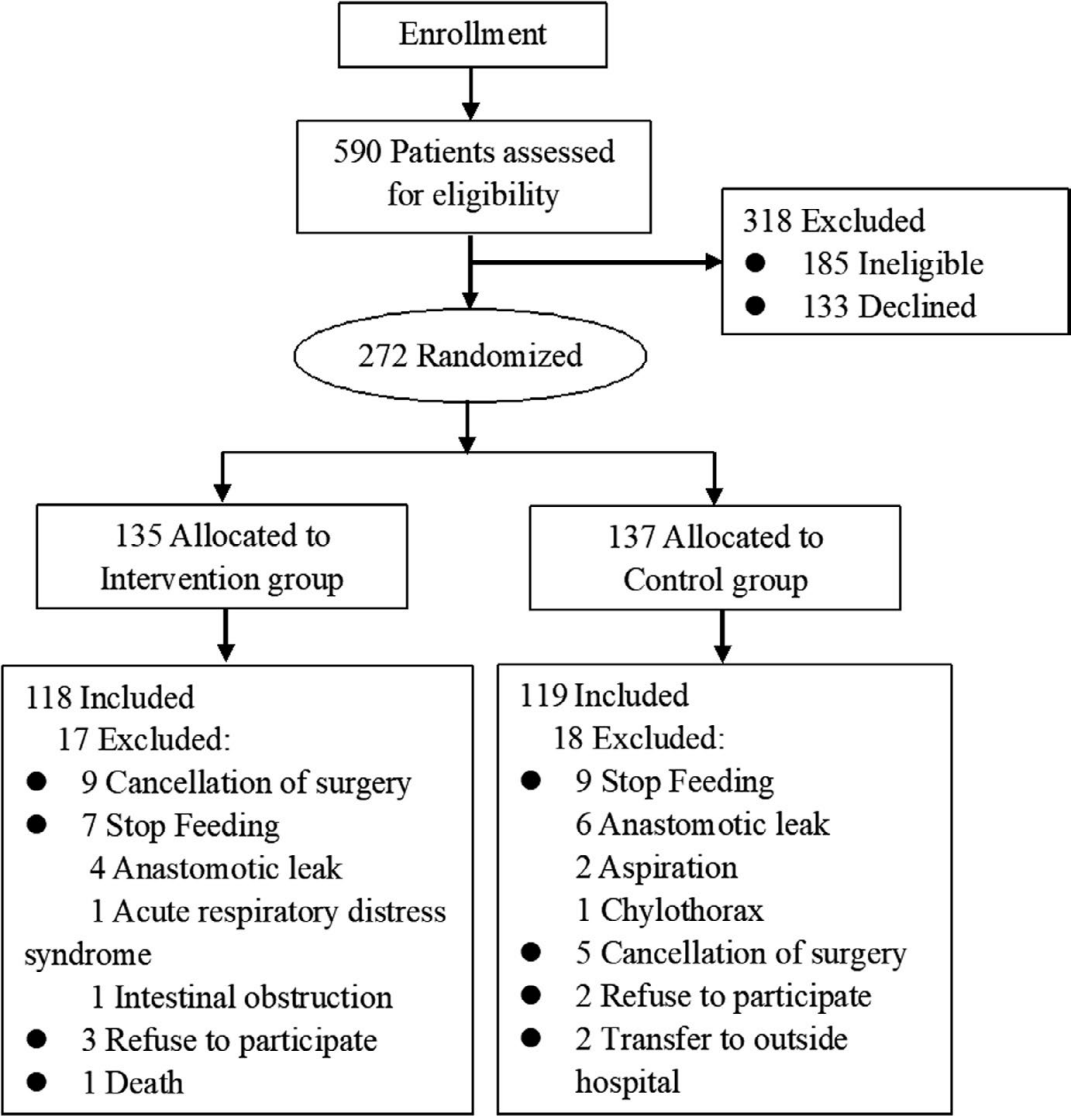
Consort diagram for the study

Of the 590 patients assessed for eligibility, 272 (46.10%) patients were included and randomized into two groups to receive the intervention of Chin‐down‐plus‐larynx‐tightening maneuver or usual care. Thirty‐five patients were excluded, and finally 237 patients were included (IG, n = 118; CG, n = 119). The detailed selection process of the participants was as shown in Figure 2. In our study, 16 patients (seven patients in the IG and nine patients in the CG) were excluded because of stop feeding, which need special explanation. Of these 16 patients, included 11 patients with anastomotic leak, 2 patients with aspiration, 1 patient with acute respiratory distress syndrome, 1 patient with intestinal obstruction, and 1 patient with chylothorax. Finally, all 16 patients recovered and were discharged with no severe adverse outcomes after treatment.

The mean (SD) age of IG was 62.79 (9.19) years, and 60.84 (10.19) years in the CG. The majority of participants were men (164/237, 69.20%), illiterate (99/237, 41.77%), married (221/237, 93.25%), unemployed (102/237, 43.04%), and middle location of tumor (119/237, 50.21%). The Pathological stage was concentrated in the Ⅱ and Ⅲ stages (190/237, 80.17%). 221 (93.25%) participants underwent bilateral lymph node resection of the recurrent laryngeal nerve. 31 (13.08%) patients showed hoarseness, 30 (12.66%) patients had pulmonary complications after operation. There was no significant difference between intervention and control groups in terms of demographic and clinical characteristics (Table 1).

**Table 1:** Demographic and clinical characteristics of patients with esophageal cancer (EC) in intervention and control groups

Variable	Intervention group (n = 118)	Control group (n = 119)	Statistics	*P* value
Age	62.79 ± 9.19	60.84 ± 10.19	1.55	.123
Gender			1.72	.190
Male	77	87		
Female	41	32		
Education			5.28	.071
Illiterate	58	41		
Sixth grade and below diploma	49	63		
Diploma and higher	11	15		
Marital status			1.19	.551
Married	108	113		
Divorced	6	4		
Widowed	4	2		
Occupation			2.20	.333
Unemployed	53	49		
Working	41	36		
Retired	24	34		
Operation time (h)	5.02 ± 0.65	4.86 ± 0.79	1.70	.090
Location of tumor			4.76	.093
Upper	23	19		
Middle	65	54		
Lower	30	46		
Pathological stage			6.05	.109
0	4	2		
Ⅰ	18	23		
Ⅱ	36	50		
Ⅲ	60	44		
Recurrent laryngeal nerve lymph node dissection			2.06	.358
Bilateral	112	109		
Left	5	6		
Right	1	4		
Hoarseness			2.75	.252
No	98	104		
Yes	19	12		
Uncertain	1	3		
Postoperative pulmonary complications			0.79	.374
No	95	101		
Yes	23	18		

### 3.2 Efficacy of effect of the Chin‐down‐plus‐larynx‐tightening maneuver

#### 3.2.1 The incidence of choking cough

The incidence of postoperative choking cough of all subjects was 19.41% (46/237). As shown in Table 2, the incidence of choking cough in the IG and the CG pre‐intervention were 21.19% and 17.65%, respectively. After implementation of the intervention, choking cough occurred in 5 of 118 cases (4.24%) in IG and 18 of 119 cases (19.4%) in CG. The incidence of choking cough within postoperative was statistically significantly lower in the IG (*P *< .05).

**Table 2:** Incidence of choking cough of patients with esophageal cancer (EC) in intervention and control groups

The incidence of choking cough	Pre‐intervention	Post‐intervention
Intervention group (n = 118)	21.19 (25/118)	4.24 (5/118)
Control group (n = 119)	17.65 (21/119)	15.13 (18/119)
Statistics	0.47	8.02
*P* value	.491	.005

#### 3.2.2 The level of swallowing function

The new swallowing maneuver was even more effective than usual care in improving swallowing function as measured by water‐drinking test. As shown in Table 3, the percentage of patients whose level of swallowing function were assessed as Ⅰ and Ⅱ, was 79.66% (94/118) in the IG, which was much higher than the CG (40.34%, 48/119). The level of swallowing function post‐intervention showed statistical different between the two groups (*P *< .001).

**Table 3:** Level of swallowing function of patients with esophageal cancer (EC) in intervention and control groups

Level of swallowing function	Pre‐intervention					Post‐intervention				
	Ⅰ	Ⅱ	Ⅲ	Ⅳ	Ⅴ	Ⅰ	Ⅱ	Ⅲ	Ⅳ	Ⅴ
Intervention group (n = 118)	5	39	48	26	0	22	72	20	4	0
Control group (n = 119)	6	29	67	17	0	10	38	59	12	0
Statistics	6.58					38.26				
*P* value	.087					<.001				

#### 3.2.3 Dietary outcomes

**Figure 3:**
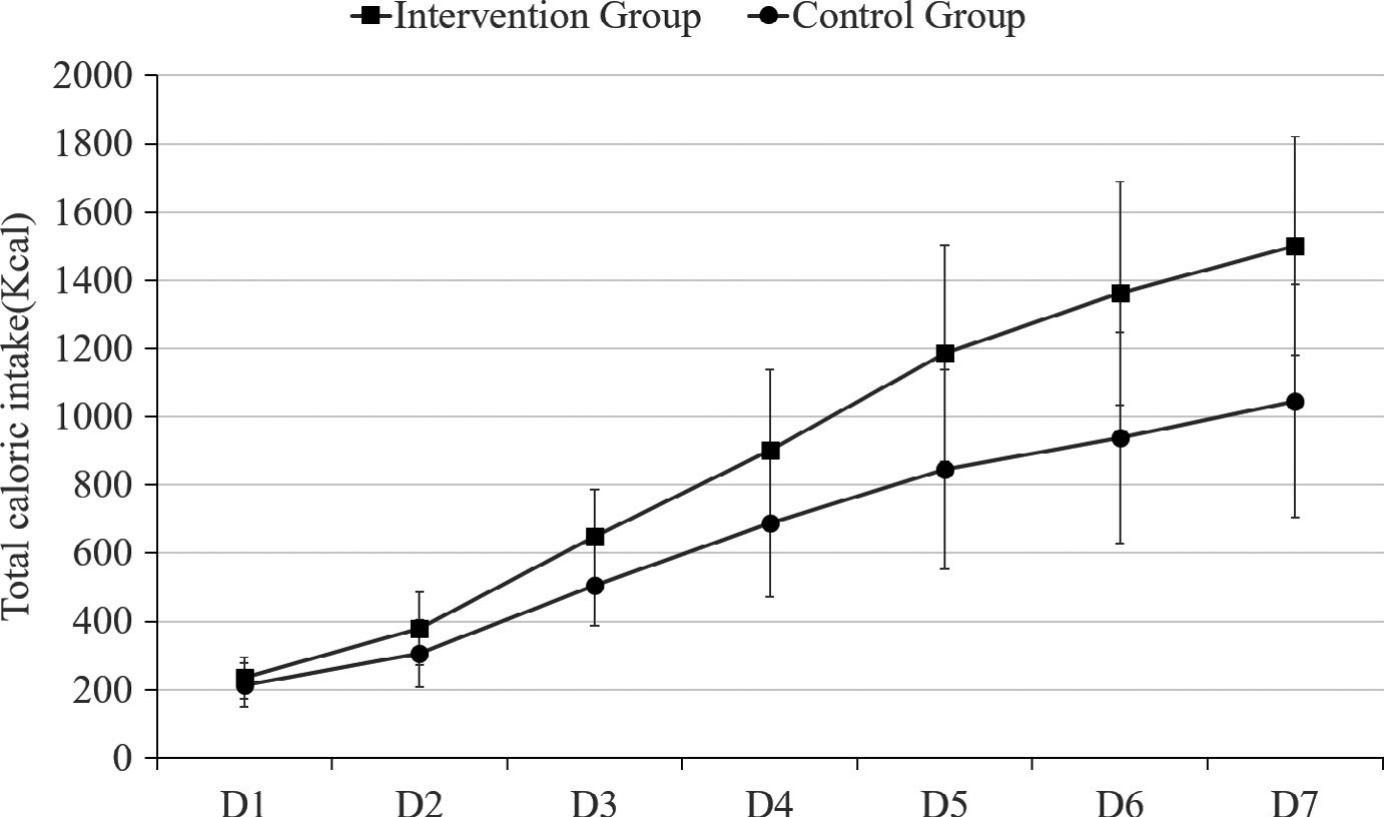
The total caloric intake of patients with esophageal cancer (EC) in intervention and control groups

**Figure 4:**
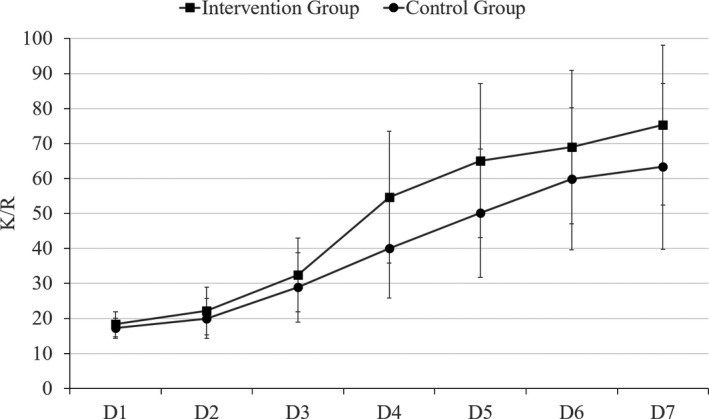
The *K*/*R* value of patients with esophageal cancer (EC) in intervention and control groups

The total caloric intake and *K/R* value of two groups were rising gradually from the first to the seventh day of intervention, as showed in Figures 3 and 4. Whereas, the dietary indicators postoperative had grown more rapidly in the IG than CG from the first day of intervention, resulting in a significant difference (*P *< .05).

## 4 DISCUSSION

Early oral intake is one of the important signs of enhanced recovery after surgery for EC patients after esophagectomy. Sun HB et al^18^, ^19^ have shown that it is safe and feasible for patients to have oral liquid diet on POD 1 following thoracolaparoscopic esophagectomy. Previous studies reported a 4%‐10% incidence of aspiration following esophagectomy, which is considered an important reason for delaying the early oral food intake nutrition.^11^, ^20^

Swallowing is a complicated process that requires coordination of multiple structures. A post‐operative choking cough accompanying feeding indicates the changes in the swallowing phases or failure of airway protection. Sasaki et al^21^ had studied six patients who had undergone an esophagectomy with postoperative abnormal aspiration episodes, and found that those cases showed weak pharyngeal contraction pressure as well as an uncoordinated response and frequent incomplete relaxation of upper esophageal sphincter. Park et al^22^ reported that when swallowing function is impaired, food residue collects in the spaces of the pharynx, which was also related to choking cough and aspiration directly. Similarly, Ahmed et al^23^ also found that the head‐turn‐plus‐chin‐down maneuver could be effective in reducing persistent vallecular residue for thin and nectar‐thick fluids.

Physiologically, cough is a coordinated series of respiratory, pharyngeal and laryngeal muscle activity, an airway protective function. The main reasons for post‐operative choking cough considered are as follows: a. The recurrent laryngeal nerve (RLN) is the main motor nerve of the larynx, which is involved in the airway protection mechanism during feeding. RLN lymph node is considered as a common metastatic site for EC and should be resected during surgery for the prognosis of esophageal cancer.^24^, ^25^ However, resection of RLN lymph node may cause RLN injuries and paralysis in 9%‐50% of cases, which can affect the glottal closing function while eating, causing water or food enter to trachea, and irritating cough and aspiration occurred.^24^, ^26^, ^27^ b. Intubation is one of the factors that induces choking cough.^6^, ^28^ The operation of MIE takes a long time, about 4–5 hours. Tracheal intubation during surgery may cause epiglottis eversion, pharyngeal paralysis, and incomplete elevation of the larynx, which will affect the coordination of the larynx muscles during swallowing. c. The position of esophagus anastomosis is in the left neck, next to the throat. If the anastomosis is narrow, or feeding too much or too fast, food may tend to flow back up into the trachea, causing choking or aspiration. Our results showed that the incidence of postoperative choking cough in patients after MIE was as high as 19.41% (46/237). Severe choking cough can cause aspiration, pneumonia, and death.^29^ Therefore, much more attention should be attached on swallowing problems after esophagectomy by medical staff.

One point needs special explanation, although for patients with swallowing disorders, drinking water is more likely to cause cough and aspiration. However, compared with dysphagia caused by central nervous system such as stroke and Parkinson's disease, the principle of oral intake for patients after esophagectomy is different, because of the most serious complication–—anastomotic leakage. To avoid the problem of food leaking into the thorax from anastomosis, our study decided to allow patients to drink water first, and closely observe the pleural drainage, and guide patients to gradually transition to diet.

The chin‐down maneuver is used to reduce the frequency of aspiration widely and is effective for various dysphagic patients. A previous Japanese article^30^ pointed out that the neck flexion maneuver resulted in significantly decreased pressure and a longer duration of lowered pressure at the upper esophageal sphincter, which is probably due to compressed the pharyngeal cavity in the anterior‐to‐posterior direction combined with consequent upper esophageal sphincter opening. Therefore, the bolus could pass through the upper esophageal sphincter easily. Gould et al^31^ also revealed that the chin‐down maneuver seems to modify the relative movement between the laryngeal muscles that perform swallowing, and the timing of swallowing. Thus, in this study we hypothesized that the posture change of Chin‐down‐plus‐larynx‐tightening maneuver would affect the post‐operative swallowing function and improve choking cough for EC patients after esophagectomy.

This randomized clinical trial showed that the Chin‐down‐plus‐larynx‐tightening maneuver was effective in improve swallowing function of patients undergoing esophagectomy. One week after the intervention, it was found that the incidence of choking in the IG was 4.24% (5/118), which was significantly lower than the CG of 15.13% (18/119) (*P* < .05). At the same time, we used the Kubota water‐drinking test to assess patients' swallowing function after one week of intervention, and found that the swallowing level in the IG was mainly concentrated in grade Ⅰ and Ⅱ (79.66%), while the swallowing level in the CG was concentrated in grade Ⅱ and Ⅲ (81.51%), which further confirmed the effectiveness of the new maneuver. The results are consistent with studies which indicated that swallowing‐related complaints improved and more safe swallowing achieved by using this new swallowing maneuver.^6^, ^14^, ^23^ Some studies also confirmed that this new maneuver could improve swallowing‐related quality of life.^32^

The typical clinical symptom of EC is progressive dysphagia, so patients often accompanied by short or long‐term malnutrition, especially after esophagectomy. Due to the resection and anastomosis of the digestive tract during surgery, patients' original eating patterns have changed. A study from China^17^ found that patients often experienced a series of gastrointestinal symptoms, such as early satiety, choking cough, swallowing difficulty, backflow, dry mouth and so on. In addition, in clinical work we often hear patients’ complains that they have the sensation of food “stuck in the throat” while eating. These negative feelings will further increase the psychological burden of eating and cause eating fear. Eventually, oral food intake nutrition would not meet the needs of body, even though intravenous nutrition was also supplemented.

This study showed that the implementation of Chin‐down‐plus‐larynx‐tightening maneuver could reduce the incidence of cough, and thereby alleviating the fear of eating caused by cough and aspiration. The analysis of this study shows that the total caloric intake of IG was significantly more than the CG (*P* < .05) from D1 to D7 of intervention. The *K*/*R* value was range from 17.21% to 75.27%, there were significant statistical differences between the two groups. The gradual recovery of food oral intake can not only improve the malnutrition of EC patient, but also help strengthen patient's confidence in enhanced recovery after surgery.^11^ However, based on the postoperative calorie intake (234.91‐1501.41 kcal in IG; 212.52‐1045.94 kcal in CG), the oral intake calories was insufficient. Parenteral nutrition, such as amino acids, fat emulsion, and glucose, should be provided via a central line to meet body's metabolic needs.

Based on the swallowing physiology and the pathogenesis of dysphagia, this study aimed to explore ways to improve dysphagia in patients with MIE by changing the head and neck posture while feeding. The results are very encouraging, indicating that a simple, noninvasive, nonpharmacologic, noneconomic intervention as the Chin‐down‐plus‐larynx‐tightening intervention is effective and feasible to improve dysphagia in EC patients after surgery.

Some limitations should be mentioned in our research. First, although this study was a prospective study with a large sample size relatively, the research objects came from a single center. Future multi‐center study should be carried out to further verify the effectiveness of the Chin‐down‐plus‐larynx‐tightening maneuver. Second, this study did not implement double‐blind method (the research staffs were aware of the interventions and randomization results) owing to the nature of the interventional research, which may lead to the overestimation of the effect of the new maneuver. Third, the 7‐day duration of the intervention was not enough, and long‐term intervention and follow‐up plan based on fixed time point were need for patients after discharged, which remains an important area for our next research.

In conclusion, this study showed that the Chin‐down‐plus‐larynx‐tightening maneuver, a free and easy procedure, was effective and feasible in relieving choking cough and promoting swallowing function for EC patients after McKeown MIE, which also could improve the nutritional status of patients to some extent. Clinical nurses play an important role in the recovery after surgery, and have the responsibility to support, advocate and educate for their patients. The example of this novel maneuver implementation may provide new ideas for nurses to think about applying more effective methods based on physiological and pathological mechanisms, to improve the quality of care and clinical problems for patients with EC.

## ETHICS APPROVAL

This study was approved by Ethics Committee of the Affiliated Cancer Hospital of Zhengzhou University/ Henan Cancer Hospital (2014xjs4), and the protocol was registered in the ClinicalTrials.gov (registration number: NCT01998230).

## CONFLICT OF INTEREST

The authors declare no conflict of interests.

## AUTHOR CONTRIBUTIONS

Funa Yang and Xiaoxia Xu participated in the design and concept of the study. Qiyun Zou, Limin Zou, Lijuan Li, and Peinan Chen participated in the data collection and enrollment of patients. Lijuan Li, Xianben Liu, and Haibo Sun were involved in literature research, data analysis, data interpretation, and writing of the report. Funa Yang, Limin Zou, and Lijuan Li participated in the interpretation of results. All authors have participated in drafting and finalizing the report.

## Data Availability

The datasets for this manuscript are not publicly available because all our data are under regulation of the Henan Cancer Hospital. Requests to access the datasets should be directed to Xiao‐Xia Xu, 330553383@qq.com.
